# The mediating role of body roundness index in the association between dietary inflammatory index and depression: evidence from the US National Health and Nutrition Examination Survey 2007–2023

**DOI:** 10.3389/fpsyt.2025.1605449

**Published:** 2025-07-15

**Authors:** Weige Duan, Jian Ma, Shanglan Qu, Jing Zhang, Min Li, Lizhu Jiang

**Affiliations:** ^1^ School of Nursing, Dali University, Dali, Yunnan, China; ^2^ School of Health and Wellness, Yunnan Technology and Business University, Songming Vocational Education New City, Kunming, China; ^3^ Department of Clinical Psychology, The Third People’s Hospital of Yunnan Province, Kunming, China

**Keywords:** dietary inflammatory index, body roundness index, depression, mediation analysis, National Health and Nutrition Examination Survey (NHANES)

## Abstract

**Objective:**

Dietary inflammatory index (DII) and body roundness index (BRI) have been reported to be independently associated with an increased risk of depression. The purpose of this study was to examine the mediating role of BRI in the relationship between DII and depression.

**Methods:**

A total of 32,210 adults were recruited from the National Health and Nutrition Examination Survey (NHANES; 2007–2023). Depression was assessed with Patient Health Questionnaire-9 (PHQ-9), DII and BRI were calculated for each participant. Weighted multivariate logistic regressions, Spearman’s correlation, and mediation analysis were performed.

**Results:**

A higher DII was significantly associated with an increased risk of depression (OR=1.68, 95% CI: 1.32–2.13, p<0.001). Compared with the lowest quintile (Q1) of BRI, individuals in the highest quintile (Q5) showed a significantly higher risk of depression (OR=1.90, 95% CI: 1.08–3.36, p=0.027). Furthermore, both DII (r=0.071) and BRI (r=0.112) were positively correlated with depressive symptoms, and DII was also positively correlated with BRI (r=0.118), all p<0.001. Of note, BRI partially mediated the relationship between DII and depression (indirect effect 0.002, 95% CI: 0.001–0.003), accounting for 10.7% of the total effect. The mediating effect of BRI was verified in both male and female population.

**Conclusions:**

This study firstly identified a mediating role of BRI in the association between DII and depressive symptoms, suggesting that visceral obesity may be an important pathway through which dietary inflammation affects depression. Our findings may provide evidence-based insights to guide targeted interventions to prevent depression at the population level.

## Introduction

Depression is a common mental disorder and a major challenge to global public health, which is characterized by depressed mood or a prolonged loss of pleasure or interest in activities (1). It is estimated that the global prevalence of depression has skyrocketed to 32% during the COVID-19 pandemic (2). The prevalence of depression among adults in the United States also increased from 8.7% in 2017–2018 to 10.6% in March 2020, and 14.4% in April 2020 (3). Without intervention, the burden of depression will continue to increase, with a decline in quality of life among those depressed, increased mortality, and significant socioeconomic costs (4). This multifactorial disorder is caused by a complex interaction between genetic susceptibility, environmental stressors such as trauma and socioeconomic inequality, and modifiable lifestyle factors including lack of exercise and poor dietary patterns (5). Early prevention by changing lifestyle factors, such as dietary factors, can reduce the public health burden associated with depressive symptoms (6).

In recent years, there has been increasing evidence highlighting the key role of diet-induced inflammation in the pathogenesis of neuropsychiatric diseases (7, 8). The Dietary Inflammatory Index (DII) proposed by Shivappa is a quantitative tool for assessing the impact of diet on inflammation by analyzing the effect of dietary components on inflammatory markers (9). DII scores are positively correlated with risk of depression (10). The risk of depression is significantly higher in the group with the highest intake of pro-inflammatory diet compared to the group with the lowest intake of anti-inflammatory diet (11, 12). Another study also suggested that a diet with pro-inflammatory characteristics is associated with an increased risk of depression (13). On the other hand, anti-inflammatory foods may be associated with a lower risk of depressive symptoms (14). However, possible mechanisms underlying the interaction between DII and depression have not been fully explored.

Previous studies have found a significant bidirectional relationship between obesity and depression (15–17). Obesity increases the risk of depression by 33% (18), partly due to the accumulation of visceral fat (19). The most commonly used indicator to assess obesity is the body mass index (BMI), which assesses “overall obesity” without considering body fat distribution (20). Waist circumference (WC) is also used to assess obesity, but it cannot distinguish between subcutaneous and visceral fat deposits (21). Visceral fat is positively associated with the severity and risk of depression in overweight or obese individuals (22). To address this gap, the Body Roundness Index (BRI) is a novel anthropometric measure that quantifies visceral fat distribution by integrating waist circumference, height and weight to construct a geometric model to quantify visceral fat distribution (23). BRI can more accurately assess body and visceral fat levels than body mass index or waist circumference (24). BRI not only predicts metabolic disorders such as diabetes (25) and cardiovascular disease (26), but is also strongly associated with depression in obese people (27). However, no studies have yet explored whether BRI can mediate the relationship between dietary inflammatory index and depression.

To fill this research gap, this study used the National Health and Nutrition Examination Survey (NHANES) dataset to address the association between the DII and depression and further clarify the mediating role of BRI in this relationship. This study helps to reveal the potential mechanism by which dietary inflammation affects depression through visceral fat distribution, providing an important evidence-based basis for public health policy formulation and clinical practice.

## Materials and methods

### Data source and participants selection

**Figure 1:**
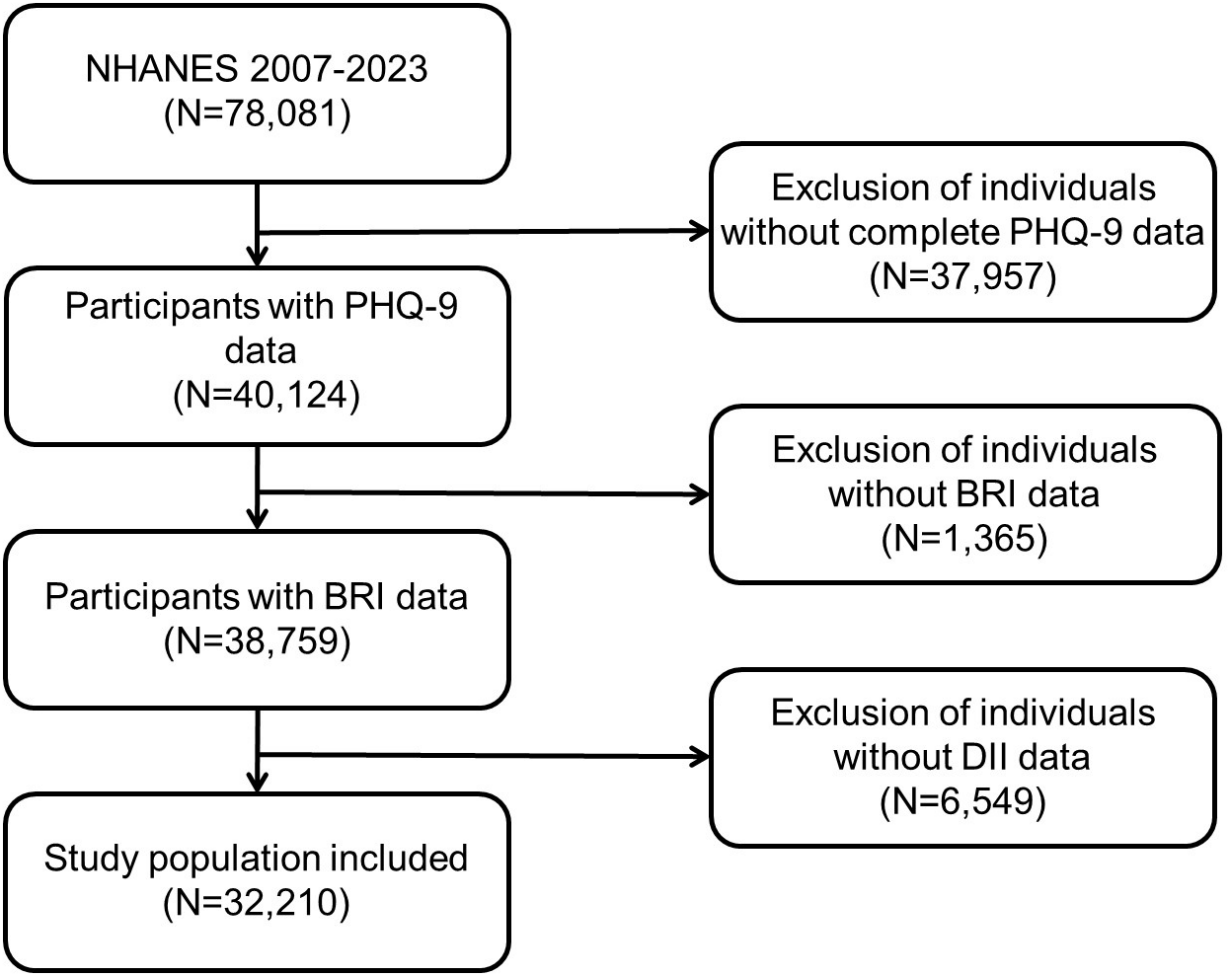
Participants’ selection process in the present study from NHANES 2007–2023. NHANES, National Health and Nutrition Examination Survey.

The NHANES is a national cross-sectional survey project conducted by the National Center for Health Statistics (NCHS) of the US Centers for Disease Control and Prevention (CDC). The objective of the NHANES is to comprehensively assess the health and nutritional status of the US population. The database covers multidimensional data, including demographics, diet, physical examination, laboratory tests, and questionnaires. It is updated every two years, with a sufficient and representative sample size, providing a rich resource for researchers. The data are publicly accessible and can be downloaded freely from the NHANES website (https://www.cdc.gov/nchs/nhanes/) without requiring special permissions. The NHANES survey protocol has been reviewed and approved by the NCHS Ethics Review Board, and written informed consent forms are obtained from all participants to ensure ethical standards are met. All personal identifiers have been removed to ensure participant anonymity. The present study incorporated a total of 78,081 subjects, encompassing seven consecutive NHANES survey cycles from 2007 to 2023. The exclusion criteria comprised individuals lacking complete PHQ-9 data (n = 37,957), individuals lacking BRI data (n = 1,365), and individuals lacking DII data (n = 6,549). The final analysis included 32,210 participants (see **Figure 1**).

### Assessment of the dietary inflammatory index

DII was calculated according to the method developed by Shivappa (9) in 2014, which associates each subject’s individual dietary data with the global average intake. The daily intake of the nutrient in question is subtracted from the global per capita daily intake of the nutrient, and then divided by the standard deviation of the global per capita daily intake of the nutrient. The z-score for each nutrient is converted to a percentage (i.e., the value ranges from 0 to 1), then symmetrically distributed around “0” by doubling and subtracting 1, and finally multiplied by the total inflammatory score for each dietary component. According to the validation of Wang et al. (28), the calculation of DII in this study includes 27 of the 45 nutrients: vitamins A/B6/B12/C/D/E, beta-carotene, iron, magnesium, zinc, selenium, caffeine, alcohol, n-3 fatty acids, n-6 fatty acids, protein, carbohydrates, cholesterol, fiber, folate, niacin, total fat, riboflavin, saturated fat, monounsaturated fat, polyunsaturated fat, and thiamin. The effect of total energy intake was controlled by standardizing the calculation of DII by adjusting it to the energy intake per 1000 kcal. The final calculation of the Energy-Adjusted Dietary Inflammatory Index (E-DII) for each individual controls for the effect of total energy intake. Individuals with an E-DII<0 are defined as having an anti-inflammatory diet, while those with an E-DII>0 are defined as having a pro-inflammatory diet.

### The assessment of depression

The Patient Health Questionnaire-9 (PHQ-9) (29) was used as a self-assessment scale for depressive symptoms that assesses the severity of depressive symptoms in an individual over the past two weeks. The responses of nine items are categorized as “not at all,” “a few days,” “more than half the days,” and “nearly every day,” and are assigned a score of 0 to 3. The total score was calculated as the sum of the scores for each item, ranging from 0 to 27. A PHQ-9 score of ≥10 is generally defined as clinically relevant depression (CRD), with higher scores indicating more severe depressive symptoms (30). The Cronbach’s α coefficient of the PHQ-9 in this study was 0.85, suggesting a high degree of internal consistency.

### The calculation of the body roundness index

BRI was calculated using a model proposed by Thomas (23). This model includes two main variables (height and waist circumference) to assess visceral fat content. A higher BRI indicates more visceral fat accumulation. The BRI is calculated using the following mathematical formula: BRI = 364.2 − 365.5 × √[1 − (waist circumference (m)/2π)^2^/(0.5 × height (m))^2^]. The quintiles of the BRI are calculated by dividing all subjects’ BRI into five groups: Q1 (1.049 ~ 3.539), Q2 (3.539 ~ 4.651), Q3 (4.651 ~ 5.770), Q4 (5.770 ~ 7.321), and Q5 (7.321 ~ 23.482).

### Covariates

According to previous studies (31–33). the study’s covariates encompassed a wide range of demographic and health-related characteristics, including age, sex, race, education level, marital status, family income-to-poverty ratio, alcohol consumption, smoking, body mass index, health status-related variables such as hypertension, coronary heart disease, and diabetes, physical activity. The age (years) of participants were divided into four groups (<24, 24–39, 40–60, and 60 or above). Ethnicity was categorized as either white or non-white, and education level was grouped as high school or less than high school. The household income-to-poverty ratio was categorized into three levels (< 1.30, 1.31 to 3.50, and > 3.50). Alcohol consumption was dichotomized as follows: having consumed at least 12 drinks in one year (yes/no). Smoking status was categorized as having smoked at least 100 cigarettes in a lifetime (yes/no). Hypertension, coronary heart disease, and diabetes were assessed based on self-reported history of disease diagnosis.

### Statistical analysis

Initially, all subjects were categorized into two groups based on their PHQ-9 scores: the depressed and the non-depressed. A normality test was conducted on all numerical variables. Those variables that conformed to a normal distribution were described as the mean (standard deviation), and two-sample t-test was used to compare the difference between groups. Non-normal variables were described as the median [interquartile range], and Mann-Whitney U test was used to compare the difference. Three weighted logistic regression models were used to explore the relationship between DII, BRI, and depression. DII was included in the model as a dichotomous variable, and BRI was included as a five-category variable. Model A did not adjust for any covariates; Model B adjusted for covariates such as sex, age, race, education, poverty, and marriage; Model C added covariates such as body mass index, alcohol consumption, smoking, hypertension, diabetes, coronary heart disease, and physical activity to model B. Spearman’s correlation analysis was used to explore the two-by-two correlation between DII, BRI, and depression. The mediating effect of BRI in the relationship between DII and depression was explored using a mediation analysis, and a 95% confidence interval (CI) was fitted using the Bootstrap method. All mediating models adjusted for all covariates. In addition, we conducted subgroup analyses in different sex (male/female) to explore the mediating effect of BRI in different sex (male, female), age (<60, >=60), and race (non-Hispanic white, other races) groups. Finally, we conducted sensitivity analyses to verify our findings using weighted logistic regression and mediation analysis after excluding individuals with missing values for any covariates. We also conducted mediating analysis with DII as the dependent variable and depression as the independent variable to further explore the mediating role of BRI in the bidirectional association between DII and depression. In accordance with NHANES analytic guidelines, all analyses incorporated two-day dietary sampling weights (WT2RD), along with clustering (SDMVPSU) and stratification (SDMVSTRA), to ensure nationally representative estimates. All statistical analyses were conducted using R (4.4.1), and the mediation analysis was conducted using the R package “mediation”. All P values were two-sided, and P<0.05 was defined as statistically significant.

## Results

### Demographic characteristics

A total of 32,210 participants were included in the study, with a median age of 50 [33.64], 15,452 (48.0%) men and 16,758 (52.0%) women, of whom 2,997 reported depressive symptoms. Compared with the non-depressed group, the proportion of patients with a pro-inflammatory diet was higher in the depressed group (p<0.001). In addition, the BRI in the depressed group was higher than that in the non-depressed group (p<0.001). Participants with depressive symptoms were mostly female, had a lower level of education, were single or separated, had a lower income level, smoked, drank, lacked exercise, and were prone to hypertension, diabetes or coronary heart disease. See **Table 1** for details.

**Table 1:** Characteristics of study participants from NHANES 2007–2023.

Characteristic	Total (N=32210)	Non-depressed (N=29,213)	Depressed (N=2,997)	P*-*value
Age (years), median (IQR)	50 (33,64)	50 (33,64)	49 (33,61)	<0.001
Age group (years), N (%)				<0.001
<24	3434 (10.7)	3120 (10.7)	314 (10.5)	
24 to 39	7598 (23.6)	6884 (23.6)	714 (23.8)	
40 to 60	10067 (31.3)	8957 (30.7)	1110 (37.0)	
≥60	11111 (34.5)	10252 (35.1)	859 (28.7)	
Sex, N (%)				<0.001
Male	15452 (48.0)	14366 (49.2)	1086 (36.2)	
Female	16758 (52.0)	14847 (50.8)	1911 (63.8)	
Race group, N (%)				0.479
White people	14346 (44.5)	13030 (44.6)	1316 (43.9)	
Non-white people	17864 (55.5)	16183 (55.4)	1681 (56.1)	
Education group, N (%)				<0.001
Above high school	24572 (80.1)	22571 (81.2)	1316 (43.9)	
Below high school	6090 (19.9)	5223 (18.8)	867 (30.2)	
Marital status, N (%)				<0.001
Coupled	18178 (59.3)	16920 (60.9)	1258 (43.9)	
Single or Separated	12485 (40.7)	10875 (39.1)	1610 (56.1)	
The ratio of family income to poverty, N (%)				<0.001
<1.30	8686 (29.6)	7389 (27.8)	1297 (48.0)	
1.3 to 3.50	10972 (37.4)	9994 (37.6)	978 (36.2)	
>3.50	9642 (32.9)	9213 (34.6)	429 (15.9)	
Alcohol drinking status, N (%)				0.047
No	7105 (22.4)	6484 (22.5)	621 (20.9)	
Yes	24658 (77.6)	22309 (77.5)	2349 (79.1)	
Smoking status, N (%)				<0.001
No	13454 (42.7)	11802 (41.3)	1308 (44.2)	
Yes	18088 (57.3)	16780 (58.7)	1652 (55.8)	
Body Mass Index, median (IQR)	28.2 (24.4,32.9)	28.08 (24.34,32.6)	30 (24.25,35.4)	<0.001
Body Mass Index group, N (%)				<0.001
<25	9042 (28.2)	8340 (28.7)	702 (23.6)	
25 to 30	10370 (32.4)	9590 (33.0)	780 (26.2)	
>30	12626 (39.4)	11136 (38.3)	1490 (50.1)	
Hypertension, N (%)				<0.001
No	20714 (64.4)	19081 (65.4)	1633 (54.5)	
Yes	11469 (35.6)	10108 (34.6)	1361 (45.5)	
Coronary heart disease, N (%)				<0.001
No	29269 (95.7)	26591 (95.9)	2678 (93.9)	
Yes	1306 (4.3)	1132 (4.1)	174 (6.1)	
Diabetes, N (%)				<0.001
No	27300 (86.9)	24983 (87.6)	2317 (80.0)	
Yes	4102 (13.1)	3522 (12.4)	580 (20.0)	
Physical activity, N (%)				<0.001
No	14377 (77.2)	12658 (75.8)	1719 (90.2)	
Yes	4239 (22.8)	4052 (24.2)	187 (9.8)	
E-DII group, N (%)				<0.001
Anti-inflammatory	8089 (25.1)	7647 (26.2)	442 (14.7)	
Pro-inflammatory	24121 (74.9)	21566 (73.8)	2555 (85.3)	
E_DII, median (IQR)	0.00075 (−0.000004,0.00175)	0.00071 (−0.00003,0.00169)	0.00119 (0.00035,0.00226)	<0.001
BRI, median (IQR)	5.18 (3.84,6.84)	5.12 (3.81,6.73)	5.96 (4.24,7.90)	<0.001
BRI group, N (%)				<0.001
Q1	6442 (20.0)	5953 (20.4)	489 (16.3)	
Q2	6442 (20.0)	6001 (20.5)	441 (14.7)	
Q3	6443 (20.0)	5957 (20.4)	486 (16.2)	
Q4	6441 (20.0)	5796 (19.8)	645 (21.5)	
Q5	6442 (20.0)	5506 (18.8)	936 (31.2)	

NHANES, National Health and Nutrition Examination Survey; E-DII, Energy-Adjusted Dietary Iness Index.

### The relationship between DII and BRI and depression

In Model A, the pro-inflammatory diet group had a 2.48-fold increased risk of depression compared with the anti-inflammatory diet group (95% CI: 2.11–2.91, p<0.001); in the partially adjusted model (Model B), the risk of depression in the pro-inflammatory diet group was still significantly higher (OR=1.98, 95% CI: 1.66–2.36, p<0.001); in the fully adjusted model (Model C), pro-inflammatory diet was significantly positively associated with the risk of depressive symptoms (OR=1.68, 95% CI: 1.32–2.13, p<0.001).

As for BRI, the risk of depression was significantly increased in those in the highest quintile (Q5) of BRI compared to those in the lowest quintile (Q1) (OR=1.96, 95% CI: 1.66–2.32, p<0.001) in Model A. The association remained significant in Model B (OR=1.70, 95% CI: 1.41–2.05, p<0.001). The risk of depression in the highest BRI group was still elevated in the fully adjusted model (OR=1.90, 95% CI: 1.08–3.36, p=0.027). See **Table 2** for details.

**Table 2:** Associations of DII and BRI with the risks of depressive symptoms^a^.

Variables	Model A			Model B			Model C		
	OR	95% CI	P-value	OR	95% CI	P-value	OR	95% CI	P-value
DII									
Anti-inflammatory	Reference			Reference			Reference		
Pro-inflammatory	2.48	2.11–2.91	<0.001	1.98	1.66–2.36	<0.001	1.68	1.32–2.13	<0.001
BRI									
Q1	Reference			Reference			Reference		
Q2	0.89	0.72–1.10	0.277	0.98	0.77–1.24	0.853	1.20	0.84–1.70	0.310
Q3	1.02	0.85–1.22	0.852	1.14	0.93–1.38	0.203	1.59	1.03–2.44	0.035
Q4	1.39	1.14–1.69	0.001	1.37	1.11–1.70	0.005	1.70	0.99–2.91	0.055
Q5	1.96	1.66–2.32	<0.001	1.70	1.41–2.05	<0.001	1.90	1.08–3.36	0.027
p-trend			<0.001			<0.001			0.028

CI, confidence interval; OR, odds ratio;DII, dietary inflammatory index; BRI, body roundness index. ^a^ The associations are presented as ORs (95% CI). The dependent variable is depressive symptoms, and the independent variables are DII and BRI. We used anti-inflammatory diet or the lowest quintile of BRI (Q1) as a reference. Model A did not adjust for any covariates; Model B adjusted for covariates such as sex, age, race, education, poverty, and marriage; Model C added covariates such as body mass index, alcohol consumption, smoking, hypertension, diabetes, coronary heart disease, and physical activity to model B.

### Spearman’s correlation

**Table 3** showed that both DII (r = 0.071, p< 0.001) and BRI (r = 0.112, p< 0.001) are significantly positively correlated with depression severity. In addition, there is also a significant positive correlation between DII and BRI (r=0.118, p< 0.001).

**Table 3:** Results of Spearman’s correlation.

Variables	DII	BRI	Depression
DII	1	0.118	0.071
BRI	0.118	1	0.112
Depression	0.071	0.112	1

All p<0.001. Abbreviations: DII, dietary inflammatory index; BRI, body roundness index.

### Mediating effect

**Figure 2:**
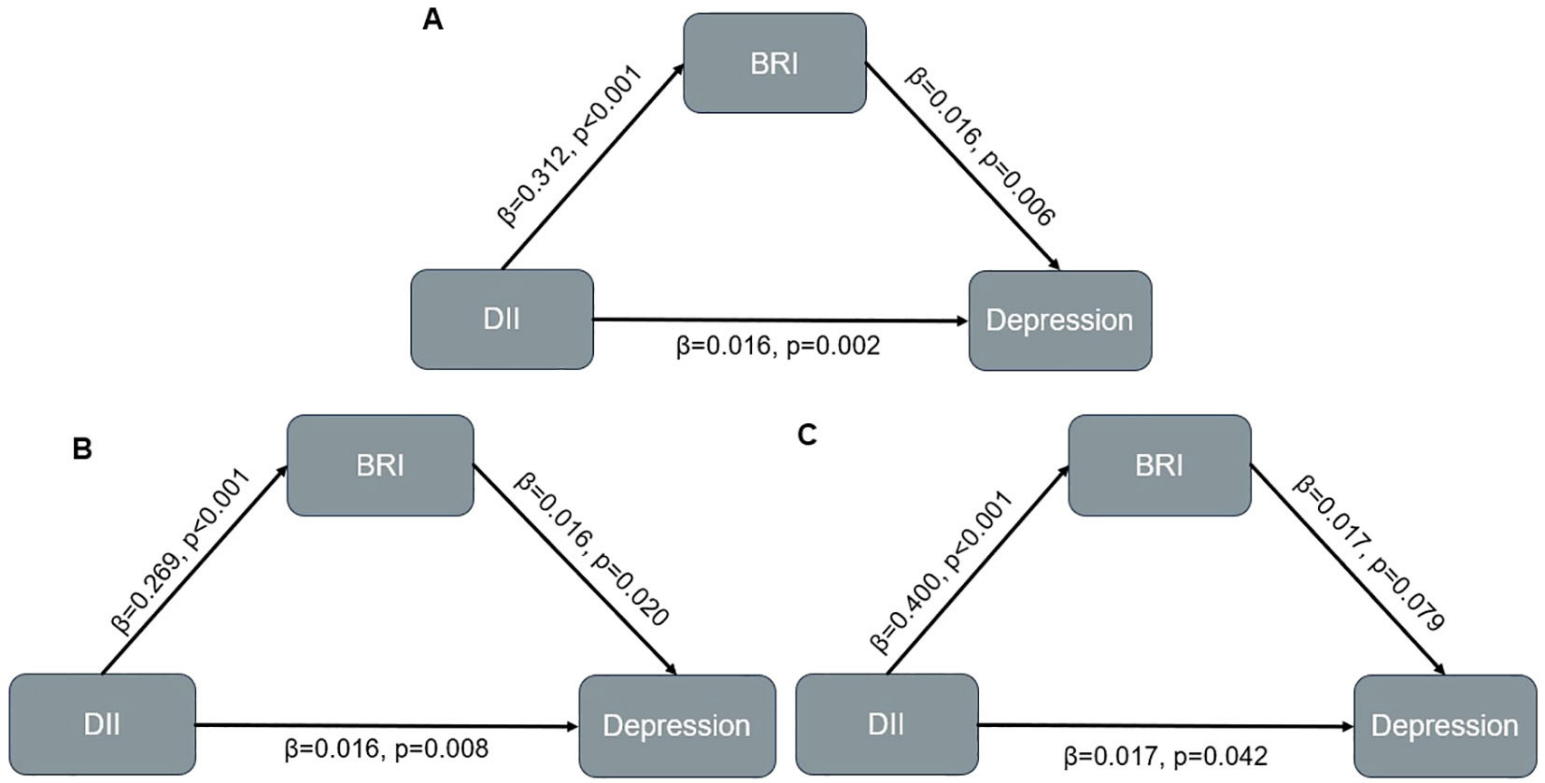
Mediation model in overall population and participants with different sex. **(A)** Overall population; **(B)** Male population; **(C)** Female population.

After adjusted for all covariates, BRI partially mediated the relationship between DII and depression (indirect effect = 0.002; 95% CI: 0.001–0.003; p<0.001), with the indirect effect accounting for 10.7% of the total effect (**Figure 2**, **Table 4**). In the male subgroup, the indirect effect was 0.002 (95% CI: 0.001–0.003, p<0.001), accounting for 9.1% of the total effect; in the female subgroup, the indirect effect was 0.002 (95% CI: 0.001–0.004, p=0.004), accounting for 11.4% of the total effect. In participants aged <60 years, the indirect effect was 0.002 (95% CI: 0.001–0.003; p < 0.001), with the indirect effect accounting for 6.9% of the total effect. Among non-Hispanic White participants, the indirect effect was 0.002 (95% CI: 0.001–0.004; p = 0.008), accounting for 8.7% of the total effect (see **Supplementary Figures 2**, **3**, **Supplementary Table 3**). No mediating analysis were further explored due to no significant total effect between DII and depression were observed in participants aged≥60 or other races.

**Table 4:** Results of mediation analyses in overall population and participants with different sex.

Groups	β	95% CI		P-value
		Lower	Upper	
Total population				
Indirect effect	0.002	0.001	0.003	<0.001
Direct effect	0.016	0.007	0.030	0.002
Total Effect	0.018	0.008	0.030	0.002
Mediated Proportion (%)	10.7%	5.0%	24.0%	0.002
Male				
Indirect effect	0.002	0.001	0.003	<0.001
Direct effect	0.016	0.003	0.030	0.008
Total Effect	0.017	0.005	0.030	<0.001
Mediated Proportion (%)	9.1%	3.1%	34.0%	<0.001
Female				
Indirect effect	0.002	0.001	0.004	0.004
Direct effect	0.017	0.001	0.030	0.042
Total Effect	0.019	0.003	0.030	0.030
Mediated Proportion (%)	11.4%	2.7%	48.0%	0.034

### Sensitivity analysis

After excluding individuals with any missing values on covariates, in the fully adjusted logistic regression model, the pro-inflammatory diet was still significantly associated with the increased risk of depressive symptoms. Compared with the anti-inflammatory diet group, the risk of depression in the pro-inflammatory diet group increased by 68% (OR = 1.68, 95% CI: 1.32–2.13, p< 0.001). Similarly, BRI was also positively associated with the risk of depressive symptoms. The risk of depression was significantly higher in individuals in the highest quintile (Q5) compared to the lowest quintile (Q1) (OR = 1.90, 95% CI: 1.08–3.36, p = 0.027) (see **Supplementary Table 1**).

In addition, the mediating analysis further confirmed that BRI also played a significant mediating role in the relationship between DII and depressive symptoms. The indirect effect was 0.002 (95% CI: 0.0011–0.0028, p < 0.001), and the mediating effect accounted for 11.0% of the total effect (see **Supplementary Table 2**, **Supplementary Figure 1**).

### Reverse mediation analysis

After adjusting for all covariates, BRI also partially mediated the relationship between depression and DII (indirect effect = 0.004; 95% CI: 0.002–0.005; p < 0.001), with the indirect effect accounting for 10.8% of the total effect (see **Supplementary Table 4**, **Supplementary Figure 4**).

## Discussion

This study is the first to reveal the relationship between DII, BRI and depression in a large nationally representative sample. We found that participants with an inflammatory diet had a higher risk of depression, and individuals with higher BRI were also more likely to be impacted by depression. In addition, BRI to some extent mediated the link between DII and depressive symptoms, and this mediating effect was statistically significant in both male and female subgroups.

The findings of this study are consistent with those of previous studies, which indicate that an elevated DII score, indicating a pro-inflammatory diet, is associated with an increased risk of depression. You et al. conducted a cross-sectional study involving 7,553 patients with metabolic syndrome and found a positive association between a higher DII and depressive symptoms (34). Moreover, Wang et al. also found in a cross-sectional study that a higher DII was significantly associated with increased risk of depression (35). Building upon these findings, Zhai et al. found in a prospective cohort study of 152,853 people based on the UK Biobank database that participants with higher dietary inflammation were more likely to develop depression (36). A significant positive correlation between the DII and depressive symptoms was also identified by Chen et al. in a systematic review (37). A case–control study of adults in Bahrain demonstrated that patients with depression exhibited a higher dietary inflammatory potential compared to controls without depression (38). In a prospective analysis of 1627 individuals, Bruno Bizzozero-Peroni et al. found that an inflammatory dietary pattern was associated with the risk of depression in older adults (39). The findings of this study support the key role of inflammation in the pathogenesis of depression, which is consistent with the neuroimmune model. According to this model, peripheral inflammatory cytokines such as TNF-α, IL-1β, and IL-6 can cross the blood–brain barrier and activate immune cells in the brain, including microglia and astrocytes (40), This activation leads to the release of more inflammatory molecules, worsening neuroinflammation (41, 42). Chronic neuroinflammation disrupts neurotransmitter systems, particularly serotonin, norepinephrine, and dopamine, by affecting their synthesis, metabolism, and receptor sensitivity (43). Additionally, sustained inflammation reduces the expression of brain-derived neurotrophic factor (BDNF), impairing neuroplasticity, especially in brain areas linked to emotion and memory, such as the prefrontal cortex and hippocampus (44). Inflammation also impacts the brain via the gut-brain axis, where gut microbiota imbalances can influence the hypothalamic–pituitary–adrenal (HPA) axis, increasing cortisol levels. Elevated cortisol exacerbates neuroinflammation, contributing to the development and progression of mood disorders, including depression (45, 46).

The present study also found that the risk of depression was higher in people with higher BRI levels, which is in line with previous works. A cross-sectional study including 18,654 adults aged 20 years and older found a positive association between BRI levels and increased depression prevalence in US adults (24). In addition, Zhang et al. also found a positive linear correlation between BRI and depression (27). However, Wang et al. found that BRI was negatively associated with depressive symptoms in a population of 11,842 elderly people aged 65 or older based on the 2018 China Longitudinal Healthy Longevity Survey (CLHLS) database (47). This is inconsistent with the results of our study, which may be related to differences in the population included in the study or the depression assessment tool. The present study’s sample is more extensive, encompassing individuals of diverse ages and ethnicities. The mean age of the participants in the present study was 50 years, whereas the mean age of the participants in Wang’s study was 83 years, thus including a more advanced age group. Age is a significant covariate that may influence the association between BRI and depression. partly due to age-related changes in adiposity distribution and inflammatory response. Indeed, previous research has shown that the association between obesity and depression tends to attenuate or become nonsignificant in older adults (48), which may partly explain the discrepancy. Secondly, this study employed the PHQ-9 to screen for depression, while Wang et al. utilized the CES-D-10, the difference in the assessment adopted may be another reason for the distinctive findings. Moreover, BRI can be situated within the conceptual frameworks of ‘allostatic load’ theory and ‘embodied health inequalities’ theory. This helps to gain a deeper understanding of how chronic stress and unhealthy lifestyles become embedded in the human body, ultimately leading to health disparities. Specifically, structural inequalities in society (e.g., poverty, racial discrimination, low levels of education) lead to long-term chronic stress by limiting access to resources (e.g., healthy diets, medical care, psychological support) (49). This stress accumulates to activate the individual’s physiological stress systems (e.g., chronic activation of the HPA axis, leading to sustained elevation of cortisol; sustained sympathetic arousal, with elevated catecholamines; and suppression of the HPG axis, with disrupted sex hormones), which in turn leads to visceral fat deposition (50, 51), which, as an active endocrine organ, is capable of secreting pro-inflammatory cytokines, such as IL-6, TNF-α, and others (52), which can cross the blood–brain barrier exacerbates neuroinflammation and impairs mood regulation in the brain (40). The BRI, as a sensitive measure of visceral fat accumulation, reflects the consequences of long-term social disadvantage exposure visualized at the biological level, which in turn effectively connects structural social inequalities to mental health outcomes.

A notable finding is that BRI partially may represent a potential pathway in the association between DII and depressive symptoms, especially in younger and non-Hispanic white population. This mediating pathway can be explained by the following biological basis: BRI is a novel indicator of visceral fat accumulation, offering a more precise quantification of visceral fat levels compared to conventional measures such as BMI and waist circumference (23). Elevated BRI is frequently associated with obesity, particularly visceral fat accumulation (53), and obesity itself has been widely confirmed to be an important risk factor for depression (54). Second, visceral fat is not only an energy storage tissue, but also a highly active endocrine organ (55). In the state of obesity, adipose tissue, particularly visceral fat, has been shown to release pro-inflammatory cytokines, such as IL-6 and TNF-α, which can lead to the activation of a systemic, chronic, low-grade inflammatory response (56). These inflammatory factors have the potential to exacerbate depressive symptoms by affecting the neurotransmitter system and activating the HPA axis (57). A higher DII score has been found to be significantly associated with an increased risk of depressive symptoms (35). A diet with high inflammatory potential contributes to the development of depressive symptoms by influencing inflammatory pathways in the nervous system, such as changes in cytokines and chemical signals (58). Among young individuals, higher DII scores are associated with greater total body fat and central obesity indicators (59, 60). Obesity, in turn, has been linked to an increased risk of depression (61). Moreover, non-Hispanic White adults exhibit higher rates of obesity and a stronger association between obesity and depression compared to other racial and ethnic groups (62). These factors may help explain the observed age and racial differences in the mediating role of BRI. On the other hand, the mediating role of BRI in the bidirectional association between depression and DII can be explained by a psychophysiological feedback loop: depression affects an individual’s mood and motivation, which leads to unhealthy eating (63) and sedentary behaviors (64). These behaviors promote the accumulation of visceral fat, which further activates pro-inflammatory responses leading to systemic inflammation (65). These pro-inflammatory factors enter the brain and trigger neuroinflammation, which in turn exacerbates depressive symptoms, creating a vicious cycle (19). Notably, the mediated proportion of BRI is relatively small, suggesting that the relationship between DII and depression share other mediating factors, for instance inflammatory biomarkers. Although the indirect effect observed in our study was relatively small (indirect effect = 0.002; 10.7% of the total effect), it was statistically significant and aligned with previous findings. For example, a prior mediation analysis showed that HOMA-IR explained 2.35% of the total effect between E-DII and depression (31). Similarly, UK Biobank studies reported that PhenoAge acceleration mediated 9.6% of the effect of E-DII on depression and 10.1% on anxiety, while KDM age acceleration accounted for 2.9% and 5.1% (13), respectively. These findings suggest that modest mediation effects are common in this field and may reflect the multifactorial biological pathways linking dietary inflammation to mental health, while small effects can translate to substantial public health impacts when scaled at the population level.

The strengths of this study include the utilization of a substantial, multi-center dataset and the implementation of systematic control for multiple potential confounders in the statistical analysis. The PHQ-9, a widely validated tool for the reliable assessment of depressive symptoms with high internal consistency, was employed in this study. Furthermore, the robustness of the results was validated through the conduct of sensitivity analyses and subgroup analyses. Based on the relationship between DII, BRI and depression, in clinical practice, anti-inflammatory diets should be guided by DII assessment, body fat control should be promoted by BRI assessment, and diet, exercise and psychotherapy should be used in an integrated manner to break the vicious cycle of depression and unhealthy lifestyles; at the policy level, food labeling laws should be implemented to assist consumers in choosing healthy foods, and public health initiatives should be implemented to focus on mental health, reduce sedentary behavior to reduce the risk of depression. The relationship between DII, BRI, and depression holds significant implications for clinical practice and policy development.

The study’s limitations include its cross-sectional design, which precludes the ability to make causal inferences regarding the relationship between DII, BRI, and depression, since the mediation model is statistical, not causal. Further validation of the causal mechanism through prospective cohort studies or intervention studies is necessary. Future longitudinal studies could examine the relationship between dietary changes and the onset of depression in greater depth, assessing diet and depression at different time points, and exploring the interaction and causality between the two factors. Additionally, the assessment of depressive symptoms relied on the PHQ-9 self-assessment scale, which may be subject to recall and reporting biases. To enhance the accuracy of the assessment, it is recommended that subsequent studies integrate clinical interviews or physician diagnoses. Thirdly, the DII is derived from two 24-hour dietary recalls, which might not completely capture the long-term dietary patterns of the subjects. Future studies should consider employing methods such as food frequency questionnaires (FFQs) to obtain more stable dietary characteristics. Due to incomplete dietary data for certain years of the National Health and Nutrition Examination Survey (NHANES), the DII calculations only included 27 food elements, which may have introduced bias into the assessment. Future studies could adopt a more comprehensive DII calculation method. Other potential confounders that were not considered include genetic susceptibility to depression, chronic psychosocial stress, unmeasured dietary patterns (e.g. eating frequency and food processing methods) and access to medication and healthcare.

## Conclusion

This study firstly suggests that elevated DII is significantly associated with the risk of depressive symptoms, with BRI playing a partial mediating role in this relationship, showing that visceral fat may be a key pathway through which dietary inflammation affects depression. This finding not only deepens the understanding of the relationship between diet, body fat distribution and mental health, but also provides novel insights for the construction of targeted prevention and intervention against depression. Improving dietary structure, reducing dietary inflammation and controlling the accumulation of visceral fat should become important directions of public health strategies to promote the mental health of the population.

## Data Availability

The datasets analyzed during the current study are publicly available from the web page of NHANES (https://www.cdc.gov/nchs/nhanes/) and can be downloaded for free.
