# Supplementary material for: The mediating role of body roundness index in the association between dietary inflammatory index and depression: evidence from the US National Health and Nutrition Examination Survey 2007–2023
Source: Front Psychiatry. 2025 Jul 15;16:1605449. doi: 10.3389/fpsyt.2025.1605449 (PMC12305813; doi:10.3389/fpsyt.2025.1605449)
Supplement: Supplementary file 1 [file SupplementaryFile1.docx]

| Supplementary Table 1. Sensitivity analysis of Associations of DII and BRI with the Risks of Depressive Symptoms ^a^. | | | | | | | | | |  |
| --- | --- | --- | --- | --- | --- | --- | --- | --- | --- | --- |
|  | Model A | | | Model B | | | Model C | | |  |
|  | OR | 95% CI | p-Value | OR | 95% CI | p-Value | OR | 95% CI | p-value |  |
| DII |  |  |  |  |  |  |  |  |  |  |
| Anti-inflammatory | Reference |  |  | Reference | |  | Reference |  |  |  |
| Pro-inflammatory | 2.45 | 1.96-3.08 | <0.001 | 1.81 | 1.44-2.27 | <0.001 | 1.68 | 1.32-2.13 | <0.001 |  |
| BRI |  |  |  |  |  |  |  |  |  |  |
| Q1 | Reference |  |  | Reference | |  | Reference |  |  |  |
| Q2 | 0.96 | 0.72- 1.29 | 0.800 | 1.10 | 0.82- 1.49 | 0.519 | 1.20 | 0.84-1.70 | 0.310 |  |
| Q3 | 1.24 | 0.91-1.68 | 0.175 | 1.36 | 1.00-1.85 | 0.203 | 1.59 | 1.03-2.44 | 0.035 |  |
| Q4 | 1.43 | 1.06-1.93 | 0.020 | 1.44 | 1.06-1.96 | 0.022 | 1.70 | 0.99-2.91 | 0.055 |  |
| Q5 | 1.95 | 1.51-2.52 | <0.001 | 1.74 | 1.33-2.28 | <0.001 | 1.90 | 1.08-3.36 | 0.027 |  |
| p-trend |  |  | <0.001 |  |  | <0.001 |  |  | 0.028 |  |
| Abbreviations: CI, confidence interval; OR, odds ratio. ^a^ The associations are presented as ORs (95% CI). Model A did not adjust for any covariates; Model B adjusted for covariates such as sex, age, race, education, poverty, and marriage; Model C added covariates such as body mass index, alcohol consumption, smoking, hypertension, diabetes, coronary heart disease, and physical activity to model B. | | | | | | | | | |  |
|  |  |  |  |  |  |  |  |  |  |  |
|  |  |  |  |  |  |  |  |  |  |  |

| Supplementary Table 2. Sensitivity analysis of mediation model summaries in the overall population. | | | | |
| --- | --- | --- | --- | --- |
|  | β | 95% CI | | p-value |
|  |  | Lower | Upper |  |
| Indirect effect | 0.002 | 0.001 | 0.003 | <0.001 |
| Direct effect | 0.015 | 0.005 | 0.020 | <0.001 |
| Total Effect | 0.017 | 0.007 | 0.030 | <0.001 |
| Mediated Proportion (%) | 11.0% | 5.3% | 26.0% | <0.001 |


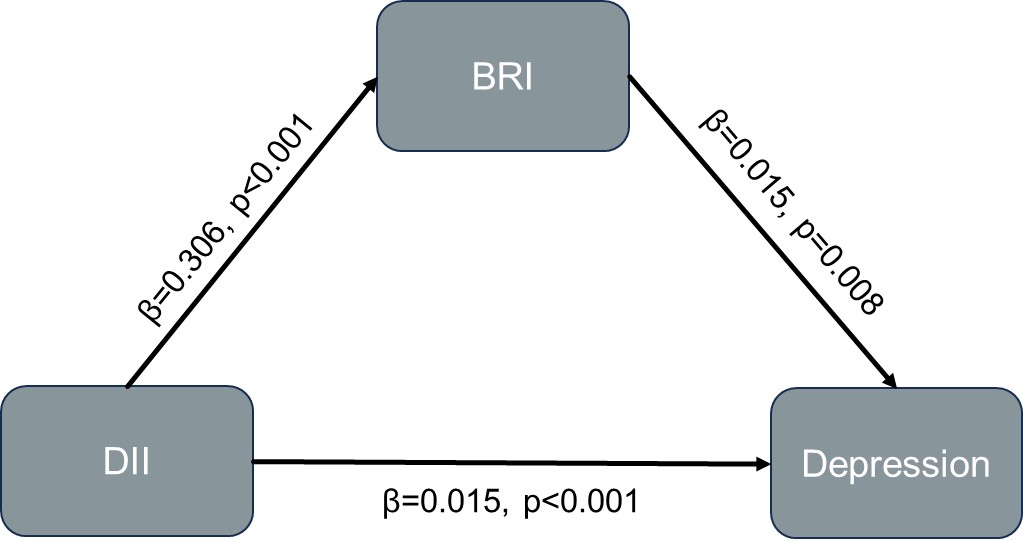


Supplementary Figure 1 Sensitivity analysis of the mediation results in the overall population.

| Supplementary Table 3. Mediation model summaries in age and race subgroups with significant total effect between DII and depression. | | | | |
| --- | --- | --- | --- | --- |
|  | β | 95% CI | | p-value |
|  |  | Lower | Upper |  |
| Aged＜60 |  |  |  |  |
| Indirect effect | 0.002 | 0.001 | 0.003 | <0.001 |
| Direct effect | 0.021 | 0.008 | 0.032 | 0.002 |
| Total Effect | 0.022 | 0.010 | 0.034 | 0.002 |
| Mediated Proportion | 0.069 | 0.029 | 0.171 | 0.002 |
| Non-Hispanic white |  |  |  |  |
| Indirect effect | 0.002 | 0.001 | 0.004 | 0.008 |
| Direct effect | 0.023 | 0.008 | 0.037 | <0.001 |
| Total Effect | 0.025 | 0.010 | 0.039 | <0.001 |
| Mediated Proportion | 0.087 | 0.023 | 0.254 | 0.008 |


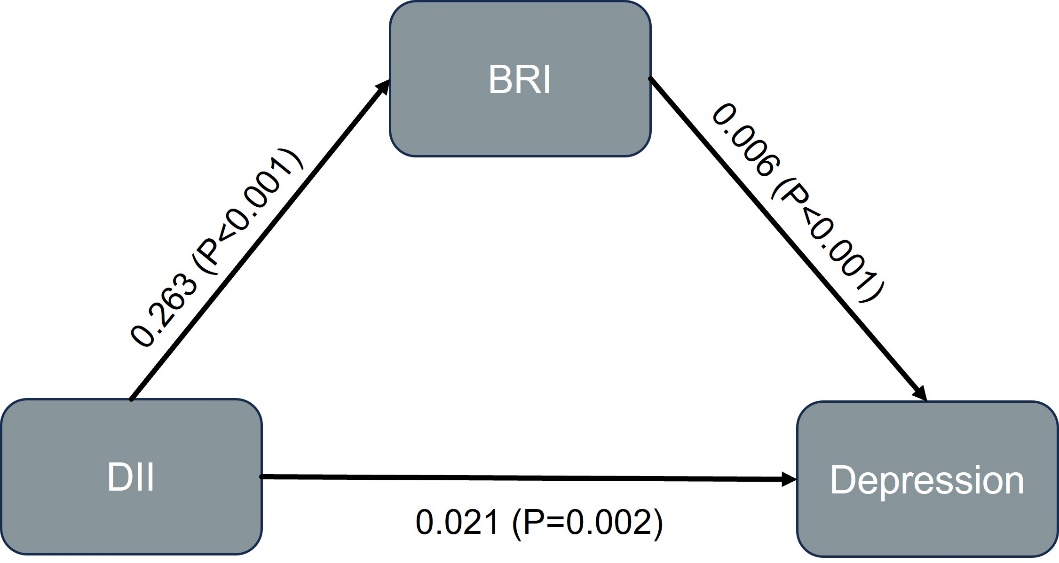


Supplementary Figure 2 Mediation results in the participants aged<60.


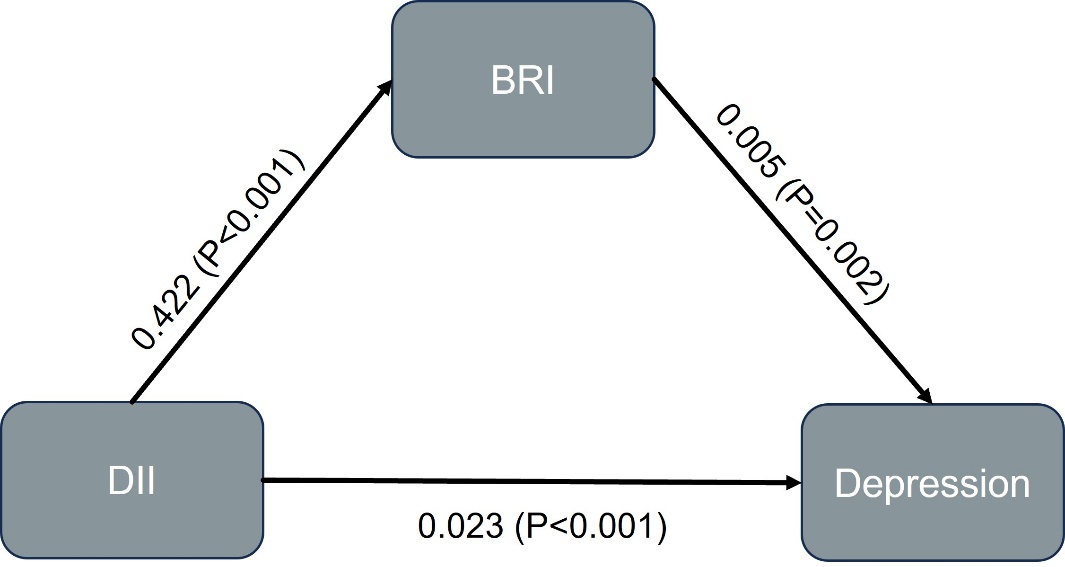


Supplementary Figure 3 Mediation results in the non-Hispanic white population.

| Supplementary Table 4. Mediation model summaries (Depression -> BRI -> DII). | | | | |
| --- | --- | --- | --- | --- |
|  | β | 95% CI | | p-value |
|  |  | Lower | Upper |  |
| Total population |  |  |  |  |
| Indirect effect | 0.004 | 0.002 | 0.005 | <0.001 |
| Direct effect | 0.030 | 0.012 | 0.049 | 0.004 |
| Total Effect | 0.034 | 0.016 | 0.053 | <0.001 |
| Mediated Proportion | 0.108 | 0.055 | 0.249 | <0.001 |


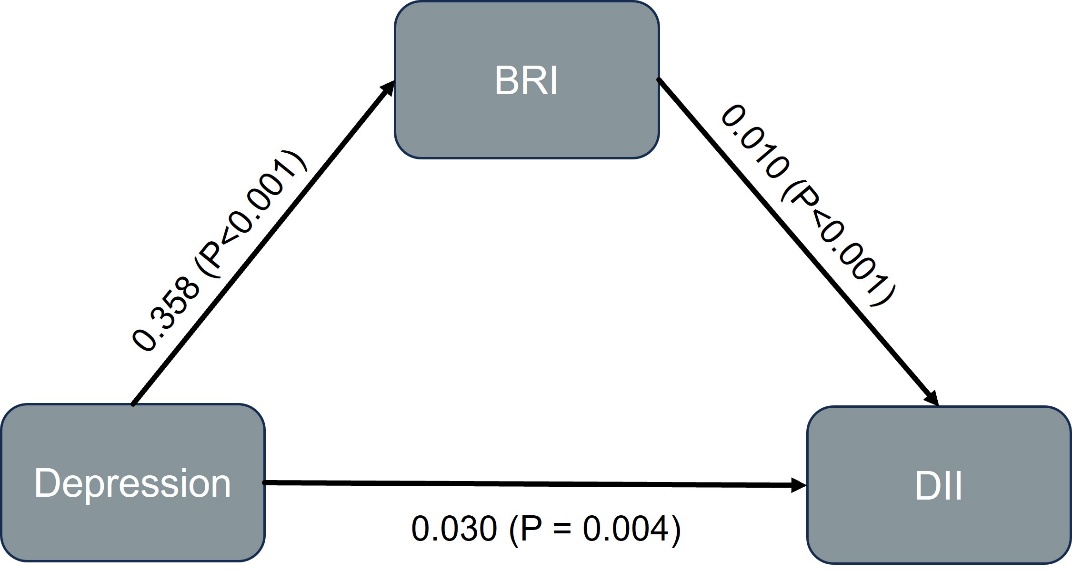


Supplementary Figure 4 Mediation results in the reversed analysis.
